# Expression of WNT Signaling Genes in the Dorsolateral Prefrontal Cortex in Schizophrenia

**DOI:** 10.3390/brainsci14070649

**Published:** 2024-06-27

**Authors:** Smita Sahay, Abdul-rizaq Hamoud, Mahasin Osman, Priyanka Pulvender, Robert E. McCullumsmith

**Affiliations:** 1 Department of Neurosciences, University of Toledo College of Medicine and Life Sciences, Toledo, OH 43614, USA; smita.sahay@rockets.utoledo.edu (S.S.); abdul-rizaq.hamoud@rockets.utoledo.edu (A.-r.H.); priyanka.pulvender@utoledo.edu (P.P.); 2 Department of Cancer Biology, University of Toledo College of Medicine and Life Sciences, Toledo, OH 43614, USA; mahasin.osman@utoledo.edu; 3 Department of Psychiatry, University of Toledo College of Medicine and Life Sciences, Toledo, OH 43614, USA; 4 Neurosciences Institute, Promedica, Toledo, OH 43606, USA

**Keywords:** schizophrenia, WNT signaling pathway, dorsolateral prefrontal cortex, gene expression, in silico, antipsychotics

## Abstract

Gene expression alterations in postmortem schizophrenia tissue are well-documented and are influenced by genetic, medication, and epigenetic factors. The Wingless/Integrated (WNT) signaling pathway, critical for cell growth and development, is involved in various cellular processes including neurodevelopment and synaptic plasticity. Despite its importance, WNT signaling remains understudied in schizophrenia, a disorder characterized by metabolic and bioenergetic defects in cortical regions. In this study, we examined the gene expression of 10 key WNT signaling pathway transcripts: IQGAP1, CTNNβ1, GSK3β, FOXO1, LRP6, MGEA5, TCF4, βTRC, PPP1Cβ, and DVL2 in the dorsolateral prefrontal cortex (DLPFC) using postmortem tissue from schizophrenia subjects (*n* = 20, 10 males, 10 females) compared to age, pH, and postmortem interval (PMI)-matched controls (*n* = 20, 10 males, 10 females). Employing the R-shiny application Kaleidoscope, we conducted in silico “lookup” studies from published transcriptomic datasets to examine cell- and region-level expression of these WNT genes. In addition, we investigated the impact of antipsychotics on the mRNA expression of the WNT genes of interest in rodent brain transcriptomic datasets. Our findings revealed no significant changes in region-level WNT transcript expression; however, analyses of previously published cell-level datasets indicated alterations in WNT transcript expression and antipsychotic-specific modulation of certain genes. These results suggest that WNT signaling transcripts may be variably expressed at the cellular level and influenced by antipsychotic treatment, providing novel insights into the role of WNT signaling in the pathophysiology of schizophrenia.

## 1. Introduction

Schizophrenia is a debilitating and widespread severe mental disorder that impacts approximately 1% of the global population [1] and is characterized by symptoms of hallucinations, delusions, low motivation, and cognitive impairments [2]. The dorsolateral prefrontal cortex (DLPFC) is a critical brain region implicated in the cognitive deficits observed in schizophrenia, showing decreased cell density, altered blood flow, and significant changes in gene expression [3,4,5,6,7,8,9,10,11,12,13,14,15,16]. The Wingless/Integrated (WNT) signaling pathway is crucial for regulating cellular processes such as proliferation, differentiation, and migration [17]. Previous studies examining gene expression changes in schizophrenia have linked higher expression of WNT signaling pathway genes to observed cortical neuronal deficits [18]. In addition, transcriptomic analyses point to a reduction in the expression of several WNT signaling genes in cortical regions as well as in whole blood samples from patients with schizophrenia [19,20]. Aside from these considerations, the WNT signaling pathway remains largely understudied in this disorder, especially in distinct, implicated brain regions such as the DLPFC. 

The WNT signaling pathways are well characterized and comprised of two main branches: the canonical WNT/β-catenin (CTNNβ1) pathway and the noncanonical pathways, which encompass the planar cell polarity pathway and the WNT/Ca^2^⁺ pathway. A pivotal protein in WNT signaling is IQ Motif Containing GTPase Activating Protein 1 (IQGAP1), which modulates the canonical and noncanonical pathways [21,22,23,24]. IQGAP1 interacts with β-catenin and disheveled segment polarity protein 2 (DVL2), influencing gene transcription and cytoskeletal formation which are critical for cellular structure and signaling [22,23]. Low-density lipoprotein receptor-related protein 6 (LRP6) is a co-receptor that facilitates β-catenin activation while glycogen synthase kinase 3 beta (GSK3β) negatively regulates the canonical WNT pathway by promoting β-catenin degradation, disruption of which has been implicated in synaptic dysfunction in the brain [25,26]. Other important regulators of WNT signaling include Forkhead box O1 (FOXO1), meningioma expressed antigen 5 (hyaluronidase) (MGEA5), transcription factor 4 (TCF4), beta-transducin repeat containing E3 ubiquitin protein ligase (βTRC), and protein phosphatase 1 catalytic subunit beta (PPP1Cβ), which collectively modulate transcriptional regulation, protein stability, and signal transduction, critical for synaptic maintenance, neurodevelopment, and overall cellular homeostasis [27,28,29,30,31]. In addition, single nucleotide polymorphisms within noncoding regions of the TCF4 locus lead to cognitive disruptions and are associated with schizophrenia [28]. Given the roles of these WNT components in signaling and metabolism and the reported bioenergetic defects in schizophrenia [18,32,33], understanding the expression patterns of WNT genes in the DLPFC may uncover potential mechanistic insights in this disorder.

In the present study, mRNA expression of WNT signaling pathway genes were assayed in the postmortem DLPFC tissue of individuals diagnosed with schizophrenia utilizing real-time quantitative polymerase chain reactions (RT-qPCR). The mRNA levels of 10 key WNT signaling genes were investigated: IQGAP1, CTNNβ1, GSK3β, FOXO1, LRP6, MGEA5, TCF4, βTRC, PPP1Cβ, and DVL2. These genes were chosen based on their integral roles in the WNT signaling pathway as well as their overlapping potential involvement in the pathophysiology of schizophrenia. The selection reflects a comprehensive approach to capturing the multifaceted involvement of WNT signaling in neurodevelopment and synaptic function, areas critically disrupted in schizophrenia. We also conducted an in silico “lookup” study utilizing published transcriptomic datasets in the Kaleidoscope software package [34] to examine cell- and region-level expression of these WNT genes. Additionally, we explored the impact of antipsychotic treatment on the mRNA expression of WNT signaling pathway genes across rodent brain transcriptomic studies to assess medication-specific effects on this pathway. This study provides greater insight into the reported altered gene expression in cortical regions of patients with schizophrenia [16,19,20] and contributes to the broader effort of deciphering the molecular pathophysiology of schizophrenia.

## 2. Methods and Materials 

### 2.1. Subjects

Postmortem human brain tissue was acquired from the Maryland Brain Collection (MBC) and the Alabama Brain Collection (ABC), with the appropriate consent obtained from the next of kin under IRB-approved protocols (IRB approval protocol number: HP-00042077). The samples comprised individuals with no prior psychiatric illness (*n* = 20) and individuals diagnosed with schizophrenia (*n* = 20). Cohorts of approximately 15–20 are standard in the field of postmortem brain studies [15,35,36,37,38]. The diagnoses were established independently by two psychiatrists through a comprehensive review of medical records, autopsy reports, and family interviews using guidelines for the Structured Clinical Interview outlined in the Diagnostic and Statistical Manual of Mental Disorders, Fourth Edition (DSM-IV), as previously described [39,40]. The specimens were collected from the DLPFC (Brodmann area 9) and were matched for age, sex, and postmortem interval (PMI, measured in hours) between the non-psychiatrically ill and schizophrenia subjects. Medication status was deemed “on” if patients were on antipsychotic medication in the last six weeks of life. Brains were partitioned in 14 μM sections on glass slides and were promptly frozen at −80 °C until required for experimentation and further analysis. Demographic information is provided in Table 1. 

### 2.2. RNA Extraction, Reverse Transcription and Complementary DNA (cDNA) Synthesis

For each subject, five 14 μM glass slide sections were scraped, and tissue was homogenized in approximately 100–150 μL of MPER protein extraction reagent (ThermoFisher Scientific, Waltham, MA, USA, Cat#: 78501) + 1X HALT protease and phosphate inhibitor (ThermoFisher Scientific, Cat#: 78429). RNA was extracted from subjects using the RNeasy isolation kit (Qiagen, Venlo, The Netherlands, Cat#: 74104) according to the manufacturer’s protocol. RNA concentrations were quantified using the Nanodrop One-C Spectrophotometer (ThermoFisher, Cat#: ND-ONEC-W) and samples were equalized to a concentration of 4.71 μg/μL using RNAse/DNAse free water. cDNA was synthesized using the High-Capacity cDNA Reverse Transcription Kit (Applied Biosystems, Carlsbad, CA, USA, Cat#: 4368814,) using 10 μL of total RNA. For the reverse transcription process, the PCR cycles comprised 1 cycle of denaturing at 95 °C for 10 min, followed by 14 cycles of denaturing at 95 °C for 14 s, and annealing at 60 °C for 4 min. Final cDNA samples were diluted 1:3 with RNAse/DNAse free water and stored at −20 °C until needed for qPCR.

### 2.3. Quantitative Real-Time Polymerase Chain Reaction (RT-qPCR)

The qPCR reactions were conducted for each subject in duplicate using 96-well optical reaction plates (Life Technologies, Carlsbad, CA, USA) on an Applied Biosystems detection system (ABI SteponePlus, Life Technologies, Carlsbad, CA, USA). Each reaction was comprised of 3 μL of cDNA in a 20 μL reaction containing 10 μL of master mix and a 1X dilution of each primer (Applied Biosystems, Life Technologies, Carlsbad, CA, USA). The TaqMan primers (Thermo Fisher, Waltham, MA, USA) for the 10 WNT signaling transcripts and two reference genes utilized in this study are detailed in Table 2. Reaction conditions involved an initial ramp time of 10 min at 95 °C, followed by 40 cycles of 15 s at 95 °C, and 1 min at the annealing temperature of 60 °C. Quality control runs examining transcript enrichment were conducted as described in previous studies [37,41]. Negative controls for the assay encompassed the omission of cDNA (non-template control) or the generation of cDNA with reverse transcriptase (RT) excluded from the reaction (no RT control). Relative concentrations of the 10 WNT transcripts of interest were computed relative to a standard curve established with cDNA dilutions from a pooled sample of all the subjects. Transcript values of interest were normalized to the geometric mean of β2M and GAPDH, reference genes with unaltered expression in the control and schizophrenia groups (Student’s *t*-test, *p* > 0.05), for all subjects in the study. 

### 2.4. In Silico “Lookup” Studies

In Silico “lookup” studies were performed using Kaleidoscope, an R shiny web application for in silico exploration of biological datasets [34]. All datasets within Kaleidoscope were curated using the National Center for Biotechnology Information Gene Expression Omnibus (NCBI GEO) genomics data repository [42,43]. 

The expressions of the 10 WNT signaling pathway genes listed in Table 2 were queried among publicly available RNA-seq and microarray-based postmortem datasets derived from humans and animal models of schizophrenia across various brain regions as well as at the neuronal cell-level. Details regarding the probed schizophrenia region-level datasets (*n* = 20) are reported in Supplementary Table S1. Details regarding the probed schizophrenia cell-level datasets (*n* = 15) are reported in Supplementary Table S2. Differential gene expression (DGE) analysis was performed across region- and cell-level datasets independently, utilizing the “Lookup” tab in Kaleidoscope. A table outlining the genes that survived correction for multiple comparisons and were significantly altered (*p* < 0.05) across datasets, along with associated log_2_-fold change (LFC) values indicating the magnitude of change between schizophrenia and control cohorts for each significant gene is detailed in Supplementary Table S3 for region-level datasets and in Supplementary Table S4 for cell-level datasets. 

Additionally, to determine the effect of antipsychotics on the expression of WNT signaling pathway genes, RNA-seq and microarray-based chronic antipsychotic-treated vs. vehicle-treated (control) rodent model datasets (*n* = 24) were probed for transcript expression changes. Details regarding the probed antipsychotic datasets are reported in Supplementary Table S5. DGE analysis was performed across antipsychotic datasets, utilizing the “Lookup” tab in Kaleidoscope. A table outlining the genes that survived correction for multiple comparisons and were significantly altered (*p* < 0.05) between antipsychotic-treated vs. control groups across datasets, along with associated log_2_-fold change (LFC) values for each significant gene is detailed in Supplementary Table S6.

The last four columns of Supplementary Tables S3, S4 and S6 provide a summary of the number of datasets all significant genes were dysregulated in specified by the direction of change as well as the average LFC values across the significantly dysregulated datasets.

### 2.5. Data Analysis

Datasets were tested for normal distribution (D’Agostino and Pearson omnibus normality test) and homogeneity of variance (F-test). Data were log transformed and were tested for outliers using the ROUT method (Q = 1%). Multiple regression analyses were conducted to determine the relationship between dependent measures (mRNA expression of 10 WNT signaling pathway genes) and age, sex, pH, and PMI. Analysis of covariance (ANCOVA) was performed in cases where significant correlations were detected. In the absence of significant correlations, an unpaired two-tailed Student’s *t*-test was performed. Bonferroni’s post hoc analyses were also conducted for all significant findings to address the issue of multiple comparisons. Lastly, a post hoc power analysis was conducted (1 − β = 0.8) to determine if the study was sufficiently powered using GPower, version 3.1 (University of Kent, Canterbury, Kent, Europe). α = 0.05 for all statistical tests. Data were analyzed using Statistica 13.0 (Statsoft, Tulsa, OK, USA) and GraphPad Prism 8.1.1 (GraphPad Software, La Jolla, CA, USA). 

## 3. Results 

### 3.1. mRNA Expression of WNT Signaling Pathway Genes

**Figure 1:**
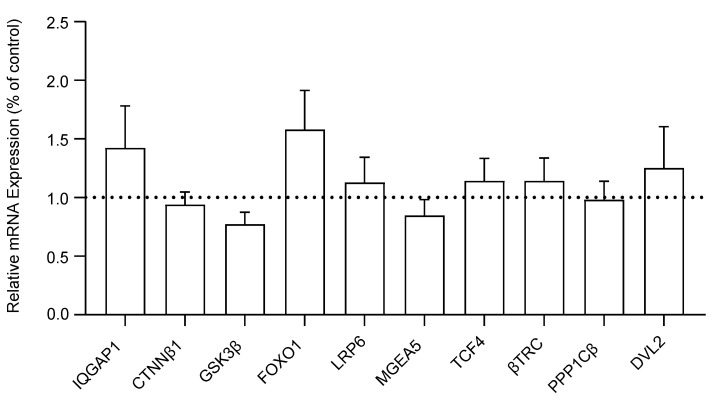
Relative mRNA expression of WNT signaling pathway genes in the dorsolateral prefrontal cortex (DLPFC) tissue of schizophrenia (*n* = 20) compared to non-psychiatrically ill control (*n* = 20) subjects, reported as percent of control (geometric mean of reference genes β2M and GAPDH). Data presented as mean ± standard error of the mean (SEM). *Abbreviations*: IQGAP1, IQ motif containing GTPase activating protein 1; CTNNβ1, Beta-catenin; GSK3β, Glycogen synthase kinase 3 beta; FOXO1, Forkhead box O1; LRP6, Lipoprotein receptor-related protein 6; MGEA5, Meningioma expressed antigen 5; TCF4, Transcription factor 4; βTRC, Beta-transducin repeat containing E3 ubiquitin protein ligase; PPP1Cβ, Protein phosphatase 1 catalytic subunit beta; DVL2, Disheveled segment polarity protein 2.

We measured and compared the mRNA expression levels of ten WNT signaling genes in the DLPFC of schizophrenia and control subjects. No significant differences were detected in the mRNA expression of IQGAP1 (F_(1, 38)_ = 1.16, *p* = 0.29), CTNNβ1 (F_(1, 38)_ = 0.17, *p* = 0.68), GSK3β (F_(1, 38)_ = 0.26, *p* = 0.61), FOXO1 (F_(1, 38)_ = 1.93, *p* = 0.17), LRP6 (F_(1, 38)_ = 0.29, *p* = 0.59), MGEA5 (F_(1, 38)_ = 0.64, *p* = 0.43), TCF4 (F_(1, 38)_ = 0.34, *p* = 0.56), βTRC (F_(1, 37)_ = 0.01, *p* = 0.92), PPP1Cβ (F_(1, 38)_ = 0.52, *p* = 0.46), or DVL2 (F_(1, 38)_ = 1.27, *p* = 0.27) in DLPFC tissue between the schizophrenia and non-psychiatrically ill control cohorts. The transcript expression of WNT genes is reported as a percent of the control reference genes (β2M and GAPDH) in Figure 1. A bar extending above the dotted line indicates that the respective WNT signaling gene is expressed at a higher percentage relative to the housekeeping genes, suggesting an upregulation in comparison to the control references (and vice versa for bars below the dotted line).

Following multiple regression analyses, no significant associations were found between the expression of any of the 10 WNT signaling pathway genes and age, sex, pH, or PMI. Since no significant differences were found in the mRNA expression of WNT genes between the schizophrenia and non-psychiatrically ill control cohorts, a post hoc power analysis was conducted to assess if our study was sufficiently powered to detect observed differences in mRNA expression between groups. The power analysis revealed that the study was sufficiently powered to detect mRNA expression differences (1 − β = 0.80, mean = 0.55, range = 0.08–1.00).

### 3.2. Region-Level Transcriptomic Results for WNT Signaling Pathway Genes

**Figure 2:**
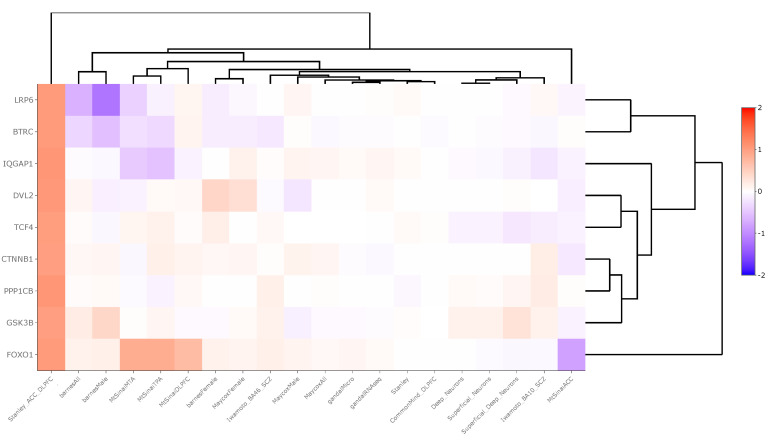
Heatmap generated from Kaleidoscope “lookup” study assessing mRNA expression of WNT signaling pathway genes across 20 RNA-seq and microarray-derived brain region-level transcriptomic datasets between schizophrenia and control groups. The heatmap utilizes unsupervised hierarchical clustering for both genes and datasets. The data are shown as log_2_-fold change (LFC) values: red indicates increased mRNA expression and blue indicates decreased mRNA expression on a scale of 2 to −2. The x-axis lists the WNT genes queried, and the y-axis lists the Kaleidoscope datasets. *Abbreviations*: IQGAP1, IQ motif containing GTPase activating protein 1; CTNNβ1, Beta-catenin; GSK3β, Glycogen synthase kinase 3 beta; FOXO1, Forkhead box O1; LRP6, Lipoprotein receptor-related protein 6; TCF4, Transcription factor 4; βTRC, Beta-transducin repeat containing E3 ubiquitin protein ligase; PPP1Cβ, Protein phosphatase 1 catalytic subunit beta; DVL2, Disheveled segment polarity protein 2.

Region-level transcriptomic datasets showed an inconsistent pattern of WNT signaling pathway gene expression between schizophrenia and control groups. Across 20 datasets, βTRC was upregulated in one dataset (LFC = 1.05) and downregulated in three (LFC = −0.13), DVL2 was upregulated in two datasets (LFC = 0.56), FOXO1 was upregulated in four datasets (LFC = 0.72) and downregulated in one (LFC = −0.82), GSK3β was downregulated in one dataset (LFC = −0.05), IQGAP was significantly upregulated in two datasets (LFC = 0.59) and downregulated in one (LFC = −0.53), LPR6 was downregulated in two datasets (LFC = −0.80), PPP1Cβ was upregulated in one dataset (LFC = 1.08), and TCF4 was upregulated in two datasets (LFC = 0.04). These data are summarized in Supplementary Table S3 and displayed in Figure 2.

### 3.3. Neuron-Level Transcriptomic Results for WNT Signaling Pathway Genes

**Figure 3:**
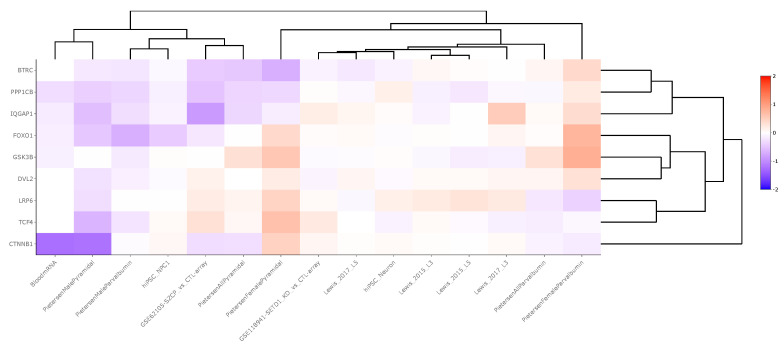
Heatmap generated from Kaleidoscope “lookup” study assessing mRNA expression of WNT signaling pathway genes across 15 RNA-seq and microarray-derived cell-level transcriptomic datasets between schizophrenia and control groups. The heatmap utilizes unsupervised hierarchical clustering for both genes and datasets. The data are shown as log_2_-fold change (LFC) values: red indicates increased mRNA expression and blue indicates decreased mRNA expression on a scale of 2 to −2. The x-axis lists the WNT genes queried, and the y-axis lists the Kaleidoscope datasets. *Abbreviations*: IQGAP1, IQ motif containing GTPase activating protein 1; CTNNβ1, Beta-catenin; GSK3β, Glycogen synthase kinase 3 beta; FOXO1, Forkhead box O1; LRP6, Lipoprotein receptor-related protein 6; TCF4, Transcription factor 4; βTRC, Beta-transducin repeat containing E3 ubiquitin protein ligase; PPP1Cβ, Protein phosphatase 1 catalytic subunit beta; DVL2, Disheveled segment polarity protein 2.

Cell-level transcriptomic datasets also showed an inconsistent pattern of WNT signaling pathway gene expression between schizophrenia and control groups, although more genes were significantly downregulated in the cell-level analysis compared to the region-level analysis. Across 15 neuronal datasets, βTRC was downregulated in one dataset (LFC = −0.45), CTNNβ1 was downregulated in two datasets (LFC = −0.78), FOXO1 was downregulated in two datasets (LFC = −0.33), GSK3β was downregulated in one dataset (LFC = −0.19), IQGAP was upregulated in one dataset (LFC = 0.54) and downregulated in one (LFC = −0.88), LPR6 was upregulated in two datasets (LFC = 0.25), PPP1Cβ was downregulated in two datasets (LFC = −0.37), and TCF4 was upregulated in one dataset (LFC = 0.33) and downregulated in one (LFC = −0.13). These data are summarized in Supplementary Table S4 and displayed in Figure 3.

### 3.4. Effect of Antipsychotics on the Expression of WNT Signaling Pathway Genes

**Figure 4:**
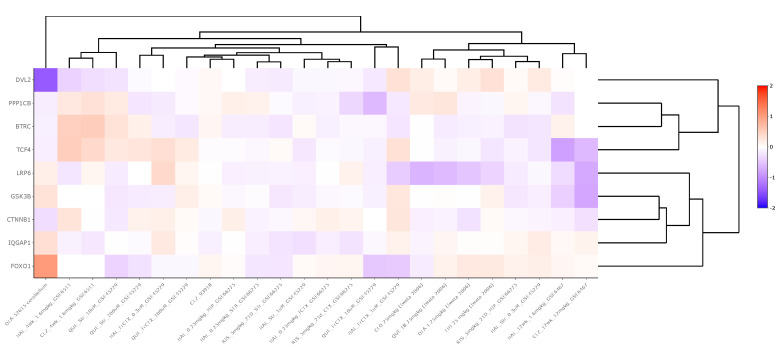
Heatmap generated from Kaleidoscope “lookup” study assessing mRNA expression of WNT signaling pathway genes across 15 RNA-seq and microarray rodent-model derived transcriptomic datasets between antipsychotic-treated and vehicle-treated (control) groups. The heatmap utilizes unsupervised hierarchical clustering for both genes and datasets. The data are shown as log_2_-fold change (LFC) values: red indicates increased mRNA expression and blue indicates decreased mRNA expression on a scale of 2 to −2. The x-axis lists the WNT genes queried, and the y-axis lists the Kaleidoscope datasets. *Abbreviations*: IQGAP1, IQ motif containing GTPase activating protein 1; CTNNβ1, Beta-catenin; GSK3β, Glycogen synthase kinase 3 beta; FOXO1, Forkhead box O1; LRP6, Lipoprotein receptor-related protein 6; TCF4, Transcription factor 4; βTRC, Beta-transducin repeat containing E3 ubiquitin protein ligase; PPP1Cβ, Protein phosphatase 1 catalytic subunit beta; DVL2, Disheveled segment polarity protein 2.

Antipsychotic datasets revealed widely variable gene expression patterns for WNT signaling pathway genes between medication and control groups. Across 24 chronically-treated (i.e., treated for a period of at least 14 days) rodent-derived datasets, βTRC was upregulated in two datasets (LFC = 0.23) and downregulated in five (LFC = −0.21), CTNNβ1 was upregulated in one dataset (LFC = 0.17), DVL2 was downregulated in one dataset (LFC = −0.20), FOXO1 was upregulated in two datasets (LFC = 0.16) and downregulated in two (LFC = −0.28), GSK3β was downregulated in three datasets (LFC = −0.20), IQGAP was upregulated in one dataset (LFC = 0.35) and downregulated in two (LFC = −0.19), LPR6 was downregulated in two datasets (LFC = −0.18), PPP1Cβ was upregulated in two datasets (LFC = 0.17) and downregulated in four (LFC = −0.35), and TCF4 was upregulated in three datasets (LFC = 0.29) and downregulated in one (LFC = −0.83). These data are summarized in Supplementary Table S6 and displayed in Figure 4.

## 4. Discussion

Our study aimed to investigate the expression of WNT signaling pathway genes in the DLPFC of individuals with schizophrenia, a region implicated in the cognitive deficits characteristic of this disorder. Despite the critical roles of WNT signaling in neurodevelopment and synaptic plasticity, our findings revealed no significant changes between schizophrenia and non-psychiatrically ill cohorts in the region-level mRNA expression of the 10 WNT signaling genes studied: IQGAP1, CTNNB1, GSK3β, FOXO1, LRP6, MGEA5, TCF4, βTRC, PPP1Cβ, and DVL2.

Previous studies have shown consistent results regarding WNT signaling in schizophrenia. For instance, studies examining WNT signaling genes in the prefrontal cortex of patients with schizophrenia reported no changes in protein expression for β-Catenin, GSK3β, and DVL2 [44]. Similarly, the mRNA expression of CTNNB1 and GSK3β remained unchanged in the DLPFC of patients with schizophrenia [45]. Our results, revealing no significant changes in the transcript expression of WNT signaling genes between schizophrenia and non-psychiatrically ill DLPFC cohorts, align with these findings. Additionally, our study focused on a wider array of signaling genes involved in the WNT signaling pathway and found no significant changes in their expression in schizophrenia, suggesting that alterations in WNT signaling at the transcript level may not be a prominent feature in the DLPFC of schizophrenia patients. 

To understand our findings further, we conducted an in silico “lookup” study assessing the mRNA expression patterns of WNT signaling pathway genes across 20 transcriptomic studies conducted between schizophrenia and non-psychiatrically ill matched control human or rodent model subjects. The samples were collected from various brain regions including the cerebral cortex, anterior cingulate cortex, temporal lobe, prefrontal cortex, and DLPFC (Supplementary Table S1). Across 20 studies, each gene was significantly dysregulated (*p* < 0.05) across *n* = 0 to *n* = 4 studies with LFC values ranging from −0.82 to 1.08. Genes were considered predominantly upregulated or downregulated in our analysis based on a ratio of at least two between upregulated and downregulated genes. Based on this criterion, βTRC and LRP6 were predominantly downregulated whereas IQGAP1, DVL2, FOXO1, and TCF4 were predominantly upregulated (Supplementary Table S3). A notable finding from this analysis was that all WNT signaling pathway genes of interested were downregulated in schizophrenia compared to control groups in the “Stanley_ACC_DLPFC” study, where data from the ACC and DLPFC brain regions were combined prior to DGE analysis (Figure 2).

βTRC, an E3 ubiquitin ligase, targets proteins for degradation, and its downregulation may result in the accumulation of proteins that disrupt cellular homeostasis [46]. LRP6 acts as a co-receptor for WNT ligands, and its reduced expression may impair WNT signaling initiation, affecting synapse formation as well as neuronal development and function [47]. On the other hand, the upregulation of IQGAP1, DVL2, FOXO1, and TCF4 points towards an adaptive or compensatory response within the WNT signaling pathway [23,28,48]. IQGAP1 and DVL2 are essential for signal transduction, and their increased expression may reflect an attempt to maintain WNT signaling activity despite other disruptions. FOXO1, involved in oxidative stress response and apoptosis, may be upregulated as a protective mechanism against cellular stress commonly observed in schizophrenia [48]. TCF4, a transcription factor, has been implicated in the regulation of genes associated with neurodevelopment and synaptic plasticity, and its upregulation may indicate a compensatory effort to modulate gene expression in response to the disorder’s pathology [28].

To further understand expression changes among WNT signaling pathway genes, we replicated our in silico “lookup” study at the cell-level, as changes in various cell types are not always detectable in region-level studies, where all cell types are mixed together [32,49]. This analysis assessed the expression of WNT transcripts across 15 transcriptomic studies conducted between schizophrenia and non-psychiatrically ill matched control subjects. The neuronal samples were collected either from induced pluripotent stem cell (iPSC) culture models or from postmortem human/rodent tissue (Supplementary Table S2). Neurons were our primary cell of interest due to the implication of pyramidal neurons in schizophrenia pathology [50]. Across 15 neuron-based studies, each gene was significantly dysregulated (*p* < 0.05) across *n* = 0 to *n* = 2 studies with LFC values ranging from −0.88 to 0.54. Similar to the region-level study conducted, genes were considered predominantly upregulated or downregulated based on a ratio of at least two between upregulated and downregulated genes. Based on this criterion, CTNNβ1, FOXO1, and PPP1Cβ were predominantly downregulated whereas LRP6 was predominantly upregulated (Supplementary Table S4). 

CTNNB1, FOXO1, and PPP1Cβ encode proteins that are crucial for WNT-mediated transcription, oxidative stress management, apoptosis, and synaptic plasticity. Reduced levels of these transcripts may increase neuronal vulnerability to damage, disrupt synaptic efficacy, and neuronal communication, impairing overall cognitive function observed in schizophrenia [51,52]. In contrast, the upregulation of LRP6 suggests a compensatory response to maintain WNT signaling, potentially enhancing pathway activation for neurodevelopment and synaptic maintenance, but it may also indicate an imbalance leading to aberrant neuronal responses. With both studies conducted, however, changes in mRNA expression may not lead to similar protein level changes [53]. Thus, further studies measuring the downstream protein activity of WNT transcripts will give further insight into functional changes in the pathology of schizophrenia.

Finally, considering that many of our datasets included data from individuals with schizophrenia who may have been taking antipsychotic medication and that these medications may impact gene expression levels [54], we wanted to examine the effect of antipsychotics on the expression of WNT signaling pathway genes. Information regarding the use of antipsychotic or over-the-counter medications by subjects prior to death may have had an impact on the mRNA expression of WNT genes (e.g., medication name, class of medication, duration of use), but this information was not readily available for use in this study. Thus, we looked up chronic antipsychotic datasets within the Kaleidoscope software package and assessed the mRNA expression of our WNT signaling pathway genes of interest across *n* = 24 rodent-derived transcriptomic studies. In each experiment, rodents were treated with antipsychotics or saline chronically (i.e., for at least a two-week period) prior to gene expression analysis (Supplementary Table S5). Across 216 gene-pathway combinations, the nine WNT signaling pathway genes were collectively significantly altered (*p* < 0.05) in approximately 32 comparisons (15%) with a wide range in the magnitude of expression change (LFC value range = −0.83 to 0.35). This finding suggests that the WNT system may be mildly modulated by chronic antipsychotic treatment at the transcript expression level; however, further sufficiently powered studies with a greater cohort of human postmortem tissue may be necessary to understand the effect of antipsychotics on WNT gene expression more precisely.

A limitation of our study is the region-level heterogeneity of the DLPFC tissue samples, which contain multiple cell types, including various glial and endothelial cells. As mentioned, this heterogeneity may mask cell-specific changes in gene expression, leading to an unchanged net expression at the region level. Future studies should isolate specific cells and perform targeted mRNA assays to analyze the expression patterns of WNT signaling genes in the DLPFC or other implicated brain regions in schizophrenia. An additional challenge is the integration of data from RNA-seq and microarray studies for the in silico “lookup” analyses. The differences in methodologies and sensitivities between these techniques may lead to variability, potentially affecting the comparability and consistency of our transcriptomic results. To mitigate noise, our data were collected from a single repository (NCBI GEO) and normalization techniques, such as batch effect correction, were implemented to harmonize data and improve the reliability and comparability of the results.

## 5. Conclusions

In summary, our study found no significant global changes in the expression of WNT signaling pathway genes in the DLPFC of schizophrenia patients at the transcript level. Furthermore, multiple regression analyses revealed no significant associations between the expression of these genes and age, sex, pH, or PMI. However, the altered expression of specific WNT pathway genes identified in previously published transcriptomic studies suggests the need for further investigation. Future research should focus on cell-specific and medication-based studies, as well as single-cell and protein activity level analyses, to better understand the role of WNT signaling in the pathophysiology of schizophrenia. These approaches will provide deeper insights into the cellular and molecular mechanisms underlying this complex disorder.

## Figures and Tables

**Table 1:** Demographics of Maryland Brain Collection and Alabama Brain Collection Subjects.

	Control	Schizophrenia
*N*	20	20
*Tissue pH*	6.6 ± 0.3	6.7 ± 0.4
*PMI (hours)*	12.6 ± 5.0	15.5 ± 6.1
*Age (years)*	42.7 ± 10.1	44.2 ± 9.4
*Sex*	10F/10M	10F/10M
*RIN*	4.5 ± 1.2	4.5 ± 1.8
*Race*	6B/14W	7B/13W
*Medication*	0/0/20	5/8/7

Data presented as mean ± standard deviation. *Abbreviations*: N, number of subjects; PMI, postmortem interval; F, female; M, male; RIN, RNA integrity; unk, unknown; RIN, RNA integrity Number; B, black; W, white.

**Table 2:** TaqMan primers.

Gene	Assay	Primer ID
*GAPDH*	Glyceraldehyde-3-phosphate dehydrogenase	Hs99999905_m1
*β2M*	Beta2-microglobulin	Hs99999907_m1
*IQGAP1*	IQ motif containing GTPase activating protein 1	Hs00896595_m1
*CTNNβ1*	Beta-catenin	Hs00355045_m1
*GSK3β*	Glycogen synthase kinase 3 beta	Hs01047719_m1
*FOXO1*	Forkhead box O1	Hs00231106_m1
*LRP6*	Low-density lipoprotein receptor-related protein 6	Hs00233945_m1
*MGEA5*	Meningioma expressed antigen 5 (hyaluronidase)	Hs01028844_m1
*TCF4*	Transcription factor 4	Hs00162613_m1
*βTRC*	Beta-transducin repeat containing E3 ubiquitin protein ligase	Hs00182707_m1
*PP1Cβ*	Protein phosphatase 1 catalytic subunit beta	Hs01027793_m1
*DVL2*	Disheveled segment polarity protein 2	Hs01005253_m1

## Data Availability

Data are contained within the article and Supplementary Tables file.

## Supplementary Materials

The following supporting information can be downloaded at: https://www.mdpi.com/article/10.3390/brainsci14070649/s1, Table S1: Information regarding the Kaleidoscope brain region-level schizophrenia datasets (*n* = 20); Table S2: Information regarding the Kaleidoscope cell-level schizophrenia datasets (*n* = 15); Table S3: Data generated from the Kaleidoscope “Lookup” study that was used to build Figure 2; Table S4: Data generated from the Kaleidoscope “Lookup” study that was used to build Figure 3; Table S5: Information regarding the Kaleidoscope chronic antipsychotic datasets (*n* = 24); Table S6: Data generated from the Kaleidoscope “Lookup” study that was used to build Figure 4.
